# A Novel Image Processing Approach to Enhancement and Compression of X-ray Images

**DOI:** 10.3390/ijerph18136724

**Published:** 2021-06-22

**Authors:** Yaghoub Pourasad, Fausto Cavallaro

**Affiliations:** 1 Department of Electrical Engineering, Urmia University of Technology, Urmia 17165-57166, Iran; 2 Department of Economics, University of Molise, Via De Sanctis, 86100 Campobasso, Italy; cavallaro@unimol.it

**Keywords:** image processing, compression enhancement, medical image, image enhancement

## Abstract

At present, there is an increase in the capacity of data generated and stored in the medical area. Thus, for the efficient handling of these extensive data, the compression methods need to be re-explored by considering the algorithm’s complexity. To reduce the redundancy of the contents of the image, thus increasing the ability to store or transfer information in optimal form, an image processing approach needs to be considered. So, in this study, two compression techniques, namely lossless compression and lossy compression, were applied for image compression, which preserves the image quality. Moreover, some enhancing techniques to increase the quality of a compressed image were employed. These methods were investigated, and several comparison results are demonstrated. Finally, the performance metrics were extracted and analyzed based on state-of-the-art methods. PSNR, MSE, and SSIM are three performance metrics that were used for the sample medical images. Detailed analysis of the measurement metrics demonstrates better efficiency than the other image processing techniques. This study helps to better understand these strategies and assists researchers in selecting a more appropriate technique for a given use case.

## 1. Introduction

Image processing is a substantial part of medical research/clinical practice [1,2]. In the last few years, medical image analysis has been highly developed by enhancing digital imaging methods. A massive number of medical images have been generated with ever-increasing diversity and quality. Although traditional medical image analysis techniques have obtained limited success, they cannot deal with colossal image data quantities [3,4,5]. The idea behind digital image processing is the processing of digital images using digital computers. Indeed, digital images are a particular composition of a limited number of elements. Each element has its location and value and is known as pixels, images, or picture elements. Generally, the term “pixel” is frequently used to point to the elements of a digital image. In medical research, medical images, e.g., CT scans, MRI, and X-ray images, are the most used images these days. Therefore, the analysis of these diverse image types requires sophisticated computerized tools. Image compression is a type of method that efficiently stores and transmits images while retaining the highest possible quality. Many software and techniques address this compression problem by establishing an appropriate balance between reconstructed image quality and compression ratio [6,7].

X-rays were discovered in 1895 by German physicist Wilhelm Roentgen who pioneered medical imaging. Medical images help physicians see through the human body, detect injuries or diseases, and direct therapeutic procedures. Based on image quality, they can determine how visible the different disease signs and anatomical structures are. The Food and Drug Administration (FDA) is an agency within the US Department of Health and Human Services, consisting of nine centers and offices. FDA has described “medical imaging” as a technology. Medical imaging includes various technologies employed to see through the human body. The primary purpose of this technology is to diagnose, monitor, or treat patients’ medical conditions. Different parts of this technology inform us about the treated or studied body area associated with a potential injury or disease or medical treatment effectiveness.

Nowadays, medical imaging technology is an essential part of medicine [8,9,10]. Surgeons, pathologists, and other medical groups can observe symptoms of the diseases directly. In recent years, medical imaging techniques have made much progress. Accordingly, planners and even surgeons can take advantage of this technology. Polap [11] displayed a versatile method composed of a hereditary calculation, and a cascade of the convolutional classifiers for picture examination was proposed. A genetic algorithm (GA) was proposed to indicate the probability of having a place for a suitable lesson. The indicated likelihood is imperative due to measuring the outcomes obtained from the cascade of neural classifiers.

This paper mainly aims to find an efficient method for compression and enhancement of the medical images. It starts with image compression and finishes with the enhancement of medical images for higher output. An in-depth study was conducted on the previous research, and various compression techniques were perused to obtain better output. Moreover, the performance metrics were extracted and analyzed based on state-of-the-art methods. Two compression techniques, namely lossless compression and lossy compression, were applied to image compression. Then, the chosen images were restored using enhancement techniques. Finally, this technique’s efficiency was analyzed using various performance parameters to assess the output. This technique was analyzed by using various performance parameters to assess the output.

The present paper is outlined as follows: Section 1 describes medical imaging and key medical imaging characteristics and quality factors. Section 2 reviews many relevant papers in medical image processing and studies some image processing methods used for improving medical images that researchers have proposed in their papers. Section 3 is the core of the present research paper. This section explains some of the significant engineering subjects related to image processing, general, and medical imaging, particularly in Section 4. The evaluation metrics are discussed in Section 5. Finally, Section 6 summarizes the numerical results and future works.

## 2. Literature Review

In this section, various image compression and image enhancement techniques are investigated. Image compression and image enhancement play a key role in medical image processing. There has been considerable research focusing on compression and image enhancement for the improvement of medical images. The Haar wavelet-based approach for image compression and quality assessment of compressed image is an image processing technique [12]. In [13], the concept of ‘Wavelet-based compression of images’ was used in grayscale images with various techniques, such as SPIHT, EZW, and SOFM. In [14], the authors deal with a specific type of compression by utilizing wavelet transforms. Wavelets were employed as simple patterns/coefficients, reproducing the initial pattern when multiplied and combined. The author of [15] introduces a new lossy compression technique that employs singular value decomposition (SVD) and wavelet difference reduction (WDR). These two methods are combined by increasing the SVD compression performance with the WDR compression.

In [16,17,18,19,20,21], the researchers investigated different steps in image processing techniques. The authors provided an overview of all relevant image processing methods, including preprocessing, segmentation, feature extraction, and classification techniques. In [22], the researchers focused their research on several medical image compression methods, such as Cosine transformations discrete, Hierarchical partitioning of the subbed block, JPEG 2000 image compression, JPEG2000 MAXSHIFT ROI coding, JPEG2000 scaling ROI coding, Mesh coding scheme, ROI-based scaling, and Wavelet adaptive shape transform. In [16,17,18,19], the researchers discussed various medical image compression techniques. A unique feature can be observed in the studied methods, but the medical images are compressed with certain drawbacks. Therefore, the research will overcome these shortcomings and increase the reconstructed quality of the compressed picture with a high compression rate for a medical image. The author introduced a new approach to image modification for visually acceptable images in [23].

The choice of image enhancement techniques depends on the particular task, picture content, viewing conditions, and observing features. Researchers have provided an overview of spatial domain techniques for image enhancement processing. More specifically, processing methods are categorized based on representative image improvement techniques. In [24], the detection of masses and segmentation techniques for image processing was studied by the authors. This study sought to use MATLAB tools in the area of medical image processing. Much medical imaging can be used in visualization tools, and many are challenging to work on. The generation by using the MATLAB package to manage and visualize matrix data will thus help create simple computer graphics, e.g., bar charts, histograms, and scatter plots.

Nowadays, image processing tool packages are available for researchers and image processing enthusiasts. The result of the proposing method has helped users to efficiently analyze and process the image in a newer software package. Wavelet-based volumetric medical image compression is provided in [25]. In this article, researchers studied how volumetric medical images can optimally be compressed using JP3D. An enhanced technique of medical compression with a lossless area is provided in [26]. Lossless techniques of compression, without any data being lost, but with a low compressive rate, and loss compression techniques with a high compression ratio but with a minor data loss can be compressed. In [27], the Medical Image Watermarking Technique for Lossless Compression is launched, which reduces the lossless watermark compression without loss of data. The watermark in this work combines the defined region of interest (ROI) and the secret key of image watermarking. An approach based on digital image compression, digital watermarking and lossless compression was presented in [28]. The authors proposed new ways of combining techniques, such as digital watermarking, image reduction/expansion, and lossless compression standards (JPEG-LS (JLS) or TIFF), amongst others. These compression techniques have been named wREPro. TIFF (Watermarked Reduction/Expansion Protocol in conjunction with TIFF) and wREPro. JLS (wREPro combined with JPEG-LS format). 

Designing convolutional neural networks’ architecture is a classic NP-hard optimization challenge, and some frameworks for creating network architectures for particular image classification tasks have been suggested [29,30]. Bacanin et al. [31] developed the hybridized monarch butterfly optimization algorithm to solve this issue. Moreover, Rosa et al. [22] used metaheuristic-driven strategies to solve the overfitting issue in the sense of CNN’s by choosing a regularization parameter known as a dropout. The findings show that optimizing dropout-based CNNs is worthwhile, owing to the ease with which appropriate dropout likelihood values can be found without setting new parameters empirically. Another method in image processing is the optimized quantum matched-filter technique [32], robust principal component analysis [33], and the generalized autoregressive conditional heteroscedasticity model [34]. Moreover, expression programming [35,36,37]. the optimization problem [38], the fuzzy best-worst method [39], and the GP-DEA model [40] are other methods. Several central math problems in medical imaging are explained in [41] by the authors.

The problem was rapidly modified by improved software and hardware. Much software is built on new techniques that utilize geometric partial differential equations combined with standard image/signal processing techniques. In this enterprise, scholars have attempted to base the principles of biomedical engineering on the development of software methods for complete rigorous mathematical foundations systems on therapy delivery. They show how mathematical research can influence some key medical subjects, such as enhancement, registration, and segmentation of the images. This research has developed an extensible image processing method that includes image compression and image enhancement to facilitate imaging research in the medical areas. Currently, it is essential to know that there is no agreement among researchers regarding image processing steps. Hence, in this paper, different compression and enhancement techniques from many researchers are studied and analyzed based on different performance metrics. At first, different MRI and CT scan images were selected, and compression methods applied to the images.

## 3. Methods and Materials

DICOM is used in nearly every radiology, cardiology, and radiotherapy imaging and radiotherapy application (X-ray, CT, MRI, ultrasound) and in equipment in other medical fields, including ophthalmology and dentistry. DICOM is one of the most commonly used healthcare communications standards globally, with hundreds of thousands of medical imaging systems in use. DICOM has revolutionized radiology practice since its inception in 1993, allowing for the complete substitution of X-ray film with a completely automated workflow. DICOM has allowed innovative medical imaging technologies that have changed the face of clinical medicine in the same way that the Internet has enabled modern customer knowledge applications. DICOM is the model that allows medical imaging work—for doctors and patients—from the emergency room to heart stress monitoring and breast cancer diagnosis.

In different image applications, wherever an image is reconstructed from its degraded version, the image processing algorithms’ efficiency needs to be measured quantitatively. For the evaluation objective, we should have the original image. In this research, medical images used to examine and evaluate methods were selected from The National Library of Medicine presents Med-Pix. Med-Pix is an online open-access database of medical images, case teaching, and clinical subjects, integrating textual metadata and images, including more than 12,000 patient cases, 9000 themes, and almost 59,000 images. The collected images are free of copyright problems and are open for use by the public. Reading some of the relevant journals and research papers, some performance parameters for evaluating image processing algorithms where the reconstructed image and the original image from its degraded version are available for evaluation objectives are listed as follows:

− Mean squared error (MSE).
− Root-mean-square error (RMSE).
− Peak signal-to-noise ratio (PSNR).
− Mean absolute error (MAE).
− Cross-correlation parameter (CP).
− Structure similarity index map (SSIM).
− Histogram analysis.

In this research, MSE, PSNR, and SSIM as performance parameters were used for evaluating image processing algorithms. Additionally, the experiments were performed using MATLAB software (MathWorks, Natick, MA, USA).

### 3.1. Image Compression Techniques

Two categories of image compression used for medical image processing research are lossless and lossy. Lossy compression requires an accurate reconstructed image of the original image from the replica. Such compression is utilized for medical image constructions, where data loss can be misdiagnosed. Unlike error-free coding, lossy image compression in exchange for a higher accuracy reduces the coded image of the compression ratio. There is a quantizer for the encoder that restricts the number of bits needed for the image. The quantizer aims to eliminate psych visual redundancy. Vector quantization, predictive coding, and transform coding are three standard methods for lossy image compression. Hybrid coding is a combined system using the characteristics of different image compression coding schemes to improve efficiency. The two ‘lossy’ techniques were used to perform a discrete cosine transform (DCT) and discrete wavelet transform (DWT). Additionally, run length encoding (RLE) and block truncation coding (BTC) of lossless techniques were applied to medical images for evaluating experiments [42].

### 3.2. Discrete Cosine Transform Technique

The discrete cosine transform (DCT) technique comprises a fixed series of data points as a total of the fluctuation of cosine functions at various frequencies [43]. In contrast to every other medical imaging technique (grayscale), DCT results show better MSE and compression ratio results. Additionally, several studies on grayscale medical images have confirmed this claim. The DCT, in comparison with other techniques, is faster than other methods for an image with smooth edges. DCTs are essential and crucial for various applications in the field of medical science/engineering. In lossy compression of audio files, such as MP3, and images, such as JPEG, the small high-frequency elements may be discarded, and DCT is suitable [43,44,45,46].

A grayscale medical image was taken from MedPix and then compressed using the DCT technique in the present research. After, inverse DCT was employed to reconstruct the medial image. This procedure was performed twice for the following reasons:

− In the first step, this work was done to reduce the image’s spatial resolution.
− In the second step, the medical image was split into blocks and re-compressed.

The first step was to do this by employing MATLAB programming, and in the next step, the image was split into blocks, and DCT was applied to each block twice.

### 3.3. Discrete Wavelet Transform Technique

Discrete wavelet transform (DWT) is a technology that enables image pixels to be transformed into wavelets and used for compression and coding on a wavelet. This technique is beneficial for compressing signals and better results for medical grayscale images [47,48]. By using the set of analysis functions, DWT enables the multi-resolution representation. In fields, such as medical imaging, the image’s degradation is not tolerated and causes a decrease in the final accuracy result. One of the best ways to extract the key information to improve the quality of signals is an approach based on using wavelets. DWT is used continuously to solve more advanced problems, providing information on the frequency and locale of the analyzed signal. Image transform from the mat to gray was done in this method and divided into 4 bits. Then, DWT compression was applied to the medical image. Finally, the image was resized to the original size again.

### 3.4. Run Length Encoding Technique

It can perhaps be said that run-length encoding (RLE) is the most straightforward common compression technique. It is a ‘lossless’ algorithm and can function by searching for ‘runs’ of the same value bits, bytes, or pixels and encrypting the run’s length and value. Therefore, RLE produces the best results with pictures with large contiguous color areas, especially monochrome pictures [49,50,51,52]. The run length encoding technique is one of the most widely used encoding methods in lossless compression techniques. It supports most bitmap file formats, such as BMP, PCX, and TIFF, and is an elementary form of lossless compression algorithms. This technique is suitable to compress any data irrespective of its content. However, the data content affects the RLE compression ratio. Without losing important information, the RLE technique can compress medical images.

Meanwhile, medical images can be compressed into a single data sequence with a long continuous sequence. Black and white images can mainly compress with run length encoding, and better results can be obtained from image compression. In the present study, lossless compression of the medical image using RLE was obtained.

### 3.5. Block Truncation Coding Technique

The technique block truncation coding is a type of grayscale lossy compression technique. In this technique, the original images are split into blocks. A quantizer is then employed to reduce the gray levels in each block with the same mean and standard differences. Much of the techniques used in RLE and BTC are used together to achieve compression outputs. BTC can also be used for video compression.

In this paper, for differentiation of the medical image into blocks in some segments, the BTC technique was used. This was achieved with the help of column altering because it can adjust total column values. It is very convenient to use the BTC technique because it can be implemented quickly, relative to other techniques in several channel errors having suitable performance.

### 3.6. Image Enhancement Techniques

Image enhancement is used to facilitate visual interpretation and imaging. Digital imagery offers the advantage that it enables us to manipulate pixel values into an image. The image enhancement technique primarily aims to modify the attributes of an image to render it more appropriate for a particular task and observation. One or more attributes of the image are changed during this process. With image enhancement methods, the interpretability or data collection in images can be enhanced for people. This method can also provide better input for other techniques of automated image processing. Nowadays, many images, such as geographic images, medical images, and aerial images, suffer from noise and poor contrast [51]. Increasing the image view’s quality, increasing contrast, blurring, and noise are the advantages of enhancement techniques. Additionally, these methods can enhance image sharpness and borders.

Two categories of enhancement techniques include:

- Spatial domain techniques.
- Frequency domain techniques.

### 3.7. Spatial Domain Methods

The primary purpose of enhancement is to process an image to yield better results for a particular process. Image enhancement is divided into two categories: spatial domain enhancement and frequency domain enhancement. The term spatial domain implies the image plane itself, which directly manipulates pixels. For the manipulation of image pixels, in this paper, the spatial domain technique was used. This method not only achieves image adjustment but can also enhance the quality and the contrast of the compressed medical images. This study used adaptive histogram equalization and morphological operations to improve the compressed medical images’ quality.

### 3.8. Adaptive Histogram Equalization (AHE)

Global histogram equalization does not work effectively for images containing low contrast regions of bright or dark areas. Adaptive histogram equalization (AHE) is the change to the histogram equalization, which can be applied for better results on these images [53]. AHE only takes small regions into account and increases the contrast of these regions by considering their local CDF. Various methods can be used to implement AHE, and there are several variations in each of those. In this project, we implemented AHE using an interpolated mapping method with tiled windows with interpolated mapping [52,54,55,56].

The medical image was enhanced with AHE by the use of MATLAB commands and functions. AHE is a method for ‘contrast enhancement’ that is widely applicable and efficient.

### 3.9. Morphological Operations (MO)

Morphological operations are easy to use and operate following the set theory. ‘Morphological operations aim to remove the ‘imperfections’ in the image structure. Most of the operations used here consist of combining two dilation and erosion processes. A small matrix structure called the structuring element is used for the operation. The shape and size of the structuring element significantly affect the final result. In image processing, morphological operations aim to remove these imperfections by considering the image form and structure [57,58,59].

### 3.10. Evaluation Metrics

An essential image processing step is medical image compression. Comparing images to evaluate the quality of compression is an essential part of measuring improvement. Metric selection is one of the challenges in evaluating medical compression [37,53]. Using the right evaluation metrics for measuring the compression and enhancement techniques is critical. Otherwise, you may be trapped in thinking that your model works well, but it does not work. We used three evaluation criteria as follows:

- Structural similarity index modulation.
- MSE.
- PSNR.

Structural Similarity Index Modulation (SSIM): The luminance, contrast, and structural are three basic computation terms used to determine the structural similarity index (*SSIM*). *SSIM* is a multiplicative combination of the three above terms:

(1) SSIM(x,y)=[l(x,y)]α  .  [C(x,y)]β  .  [S(x,y)]γ

where:

(2) $$l\left(x,y\right)=\frac{2\mu_{x}\mu_{y}+C_{1}}{\mu_{x}^{2}+\mu_{y}^{2}+C_{1}}$$

(3) $$C\left(x,y\right)=\frac{2\delta_{x}\delta_{y}+C_{2}}{\delta_{x}^{2}+\delta_{y}^{2}+C_{2}}$$

(4) $$S\left(x,y\right)=\frac{\delta_{xy}+C_{2}}{\delta_{x}\delta_{y}+C_{2}}$$

In the equations above, $\mu_{x},\;\mu_{y},\;\sigma_{x},\;\sigma_{y}$, and $\sigma_{xy}$ represent the local mean, SD, and cross-covariance for images x, y, respectively. If α=β=γ=1 (as the default values for the exponents), and $C_{3}=C_{2}/2$ (as the default value for $C_{3}$), the index can be simplified as follows:

(5) $$SSIM\left(x,y\right)=\frac{\left(2\delta_{x}\delta_{y}+C_{1}\right)\left(2\delta_{x}\delta_{y}+C_{2}\right)}{\left(\delta_{x}^{2}+\delta_{y}^{2}+C_{1}\right)\left(\delta_{x}^{2}+\delta_{y}^{2}+C_{2}\right)}$$

This method is used to evaluate the similarity between the two images. It has also been developed to improve techniques like MSE and PSNR.

Mean Squared Error (MSE): A model evaluation metric mostly applied with regression models is the mean squared error. To evaluate the compression techniques and enhancement techniques, the *MSE* method can be used:

(6) $$MSE=\frac{1}{MN}\overset{M}{\underset{y=1}{\sum }}\overset{N}{\underset{x=1}{\sum }}{\left(I\left(x,y\right)-I^{\prime }\left(x,y\right)\right)}^{2}$$

In the equation above, *I*(*x*; *y*) and *I*0(*x*; *y*) denote the original and recovered pixels’ values at row *x* and column *y* for the *M* × *N* image, respectively.

Peak Signal-to-Noise Ratio (PSNR): The reconstruction (*PSNR*) is one of the appropriate quality assessment criteria for medical image compression for medical image enhancement and peak signal-to-noise ratio (*PSNR*). PSNR indicates a ratio of the maximum possible value (power) of an indicator with the performance of a distorting noise, which generally impacts its representation quality:

(7) $$PSNR=20\log\frac{255}{\sqrt{MSE}}$$

## 4. Results

A medical image was chosen from the MedPix^®^ database to show the results. After compression and enhancement were are applied to the sample medical images, specific outputs from each image were obtained and analyzed. MedPix^®^ is an open-access online dataset of restorative pictures, educating cases, clinical subjects, coordination pictures, and printed metadata counting over 12,000 understanding case scenarios, 9000 subjects, and about 59,000 pictures. It essentially targets a group of onlookers and incorporates doctors and medical attendants, associated wellbeing professionals, medical understudies, nursing understudies, and others inquisitive about therapeutic knowledge. The substance fabric is organized by malady area (organ framework), pathology category, quiet profiles, picture classification, and picture captions. The collection is searchable by understanding side effects and signs, determination, organ framework, picture methodology, picture depiction, catchphrases, contributing creators, and numerous other look alternatives. The values take from this medical image are also categorized in Table 1.

### Enhanced and Compressed Output

**Figure 1:**
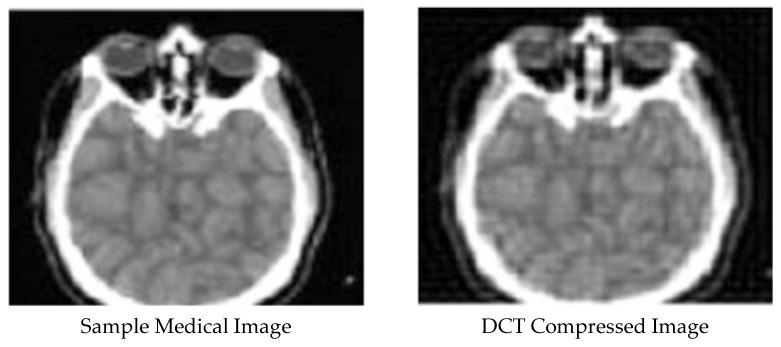

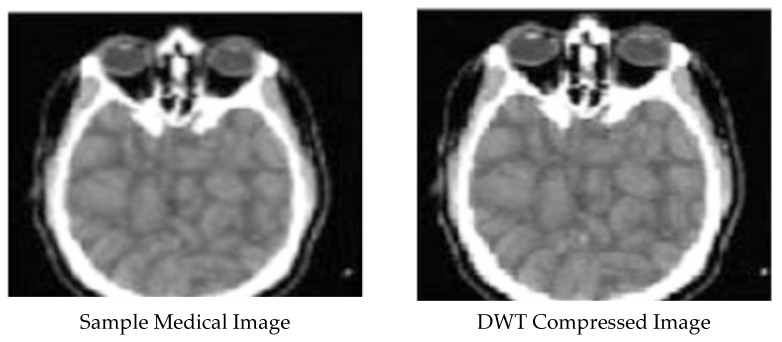
Lossy techniques: DCT and DWT compression.

**Figure 2:**
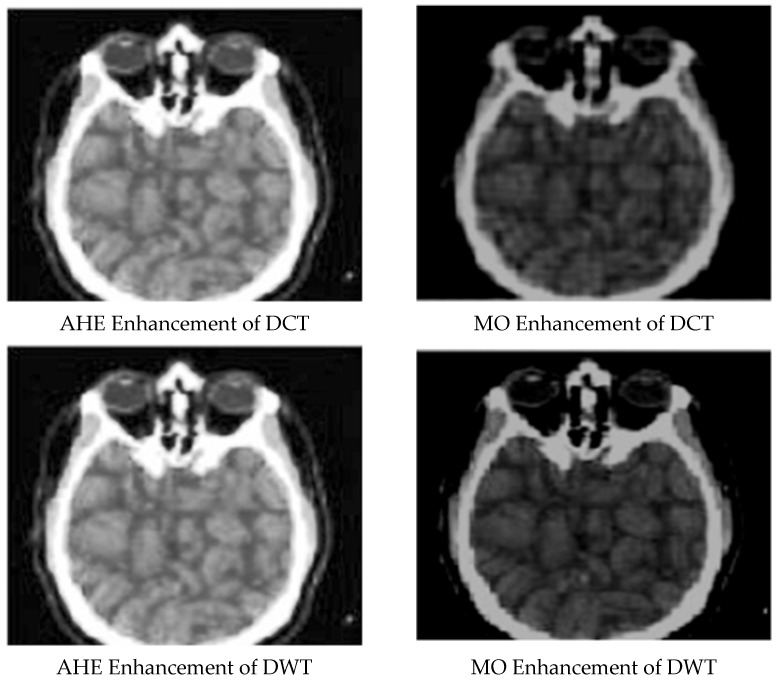
Lossy techniques: AHE and MO Enhancement for DCT and DWT.

**Figure 3:**
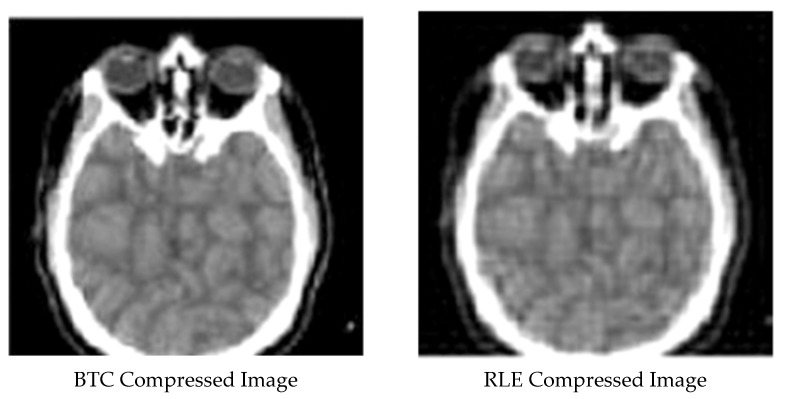
Lossless techniques: Lossless compression utilizing RLE and BTC.

**Figure 4:**
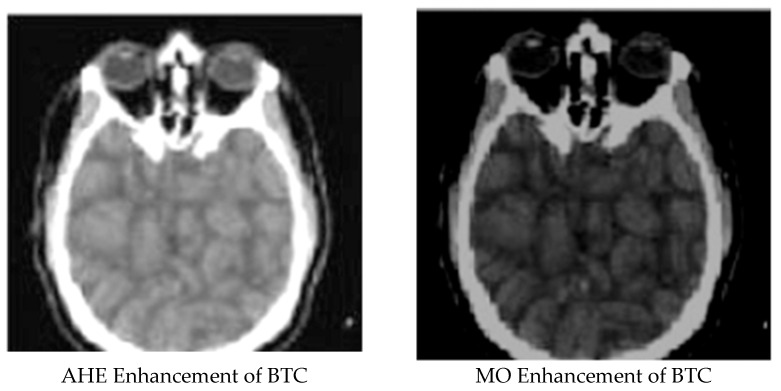
Lossless techniques: Enhancement of BTC using AHE and MO.

**Figure 5:**
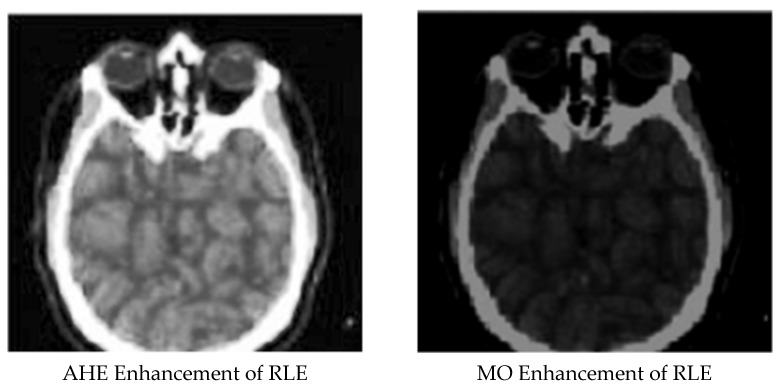
Lossless techniques: Enhancement of RLE using AHE and MO.

Figure 1, Figure 2, Figure 3, Figure 4 and Figure 5 display the enhanced and compressed output of the sample medical image.

## 5. Discussion

Image compression is an application of information compression on digital images; in other words, the purpose of this work is to reduce the redundancy of the contents of the image for the ability to store or transfer information in optimal form. Photo compression can be done without loss and total loss. Lossless compression is sometimes preferred for some images, such as technical drawings and icons, so high-loss compression methods compromise image quality, primarily when used for low bit rates. Lossless compression methods may also be preferred for valuable content, such as medical photographs or scanned photographs for archiving purposes. The proliferation method is especially suitable for natural photographs, such as photographs for small (sometimes minor) applications, where the loss of fidelity is significant to reduce the bit rate. To store images, the amount of information must be reduced as much as possible, and the basis of all compression methods is the exclusion of parts of information and data. It is the compression ratio that determines the amount and percentage of information discarded. This method simplifies data storage and transmission and reduces the required bandwidth and frequency. PSNR, MSE, and SSIM are three performance metrics that were used for the sample medical images. As shown in Table 1, to improve the images’ quality after compression with lossless and lossy techniques, morphological operations is not a suitable algorithm. By further examining and comparing the values of PSNR with the morphological operations algorithm, we found that MO is not an appropriate algorithm to enhance images after compression. In general, it can be stated that evaluation metrics values include SSIM and PSNR after MO and AHE methods are less than PSNR and SSIM values after compression; therefore, these two methods, namely AHE and MO methods, are not suitable for medical image enhancement.

**Figure 6:**
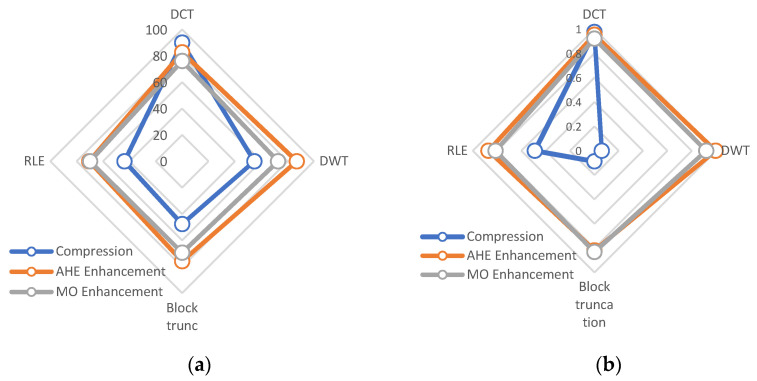
The performance of the presented methods (**a**): PSNR criteria, (**b**): SSIM criteria.

Regarding the results of the presented method in Figure 6, the graph illustrates the performance methods for both compression and enhancement. Based on our findings, the DCT method has higher PSNR than other methods and is compatible with compression. Moreover, the enhancement of the AHE method represents higher performance than other methods. Moreover, the SSIM methods indicate that the DWT and block truncation ability to compress X-ray images is weaker than the DCT and RLE techniques. After comparing the presented methods with the state-of-the-art image compression approaches, it can be estimated that the presented techniques have higher accuracy than other methods. Moreover, regarding Table 2, the lower MSE belongs to the presented DCT and RLE. Moreover, the PSNR criteria are 89.98 and 54.77 for DCT and DWT. These are higher values in comparison with those in the literature methods. 

## 6. Conclusions

This paper mainly aimed to obtain an efficient medical image output. For this purpose, a comprehensive literature review was conducted to comprehend these methods’ different features and functions. A piece of explicit knowledge was acquired on the enhancement and compression methods from this literature research. Additionally, how they work on medical grayscale images was investigated.

In the first step, compression was performed by employing both lossless and lossy methods, followed by enhancement. Four techniques, including BTC, DCT, DWT, and RLE, were applied for compression. Lossy compression using DWT enhancement based on MSE, SSIM, and PSNR without the loss of more information showed better results than the DCT technique. Without losing much data, the RLE and BTC techniques compressed well. The RLE technique compared with the BTC technique presented a reasonable compression rate from the analysis. Using the two techniques AHE and MO, each compression technique was further enhanced. Besides, the results of the analysis showed that the combination of compression and enhancement techniques works together well. Compared to PSNR and SSIM, the RLE technique showed higher values and better image quality following enhancement than the BTC technique. The experiments showed that when we combined AHE and RLE techniques, these two techniques presented more satisfactory enhancement results than the other techniques. The AHE technique considerably improved the compressed image in the DWT compression technique. Morphological operations were used instead of sharpening or increasing contrast images to enhance the background. Morphological operations werre utilized to improve the quality of the background rather than to sharpen the image. Such techniques, in particular, were used to improve the particular region of interest.

Medical imaging is rapidly developing due to the development of image processing techniques, including image recognition, testing, and improvement. Image processing increases the percentage and number of problems detected. For future work, machine learning algorithms, including supervised, unsupervised, reinforcement algorithms, meta-heuristic algorithms, approximate algorithms, and deep learning algorithms, are techniques that can be applied to image processing and image optimization with different parameters.

## Figures and Tables

**Table 1:** Performance metric for the sample medical image.

Index		SSIM	MSE	PSNR
1	DCT compressed Image	0.976102286	4.15 × 10^−5^	89.97979612
2	AHE enhancement for DCT compressed image	0.953323544	0.01256723	82.67223261
3	DWT compressed image	0.060732271	0.555070285	54.76541543
4	AHE enhancement for DWT compressed image	0.996540627	4.14 × 10^−5^	86.98046733
5	MO enhancement for DWT compressed image	0.919275875	0.012887986	72.76504215
6	Block truncation compressed image	0.087685188	0.66648485	47.63287631
7	AHE enhancement for block truncation image	0.819149803	0.001717444	75.77798049
8	MO enhancement for block truncation image	0.832122574	0.002987536	69.33196602
9	RLE compressed image	0.48970346	0.299972986	43.72625764
10	AHE enhancement for RLE compressed image	0.87101654	0.000629014	70.50874365
11	MO enhancement for RLE compressed image	0.81224123	0.001335176	69.85366112

**Table 2:** The comparison between the presented methods and the state-of-the-art.

Method	MSE	PSNR
Presented DCT	4.15 × 10^−5^	89.98
Presented DWT	0.56	54.77
Presented Block truncation	0.67	47.63
Presented RLE	0.30	43.73
D-CNN [44]	1.40	47.40
BTOT [44]	2.81	45.90
JPEG [44]	6.82	43.97
JPEG2000 [44]	1.60	47.11

## Data Availability

The dataset is available online: http://www.med.harvard.edu/AANLIB/ (accessed on 18 April 2021).

## Abbreviations

CT	Computerized Tomography
MRI	Magnetic Resonance Imaging
FDA	Food and Drug Administration
SPIHT	Set Partitioning in Hierarchical Trees
EZW	Embedded Zero Trees of Wavelet Transforms
SOFM	Self-Organizing Feature Map
SVD	Singular Value Decomposition
WDR	Wavelet Difference Reduction
ROI	Region Of Interest
CNN	Convolutional Neural Network
GP	Goal Programming
DICOM	Digital Imaging and Communications In Medicine
JPEG	Joint Photographic Experts Group
TIFF	Tagged Image File Format
BMP	Bitmap
PCX	Picture Exchange
CDF	Cumulative Distribution Function
*MO*	Morphological Operations
*AHE*	Adaptive Histogram Equalization
DCT	Discrete Cosine Transform
DWT	Discrete Wavelet Transform
RLE	Run Length Encoding
BTC	Block Truncation Coding
MSE	Mean Squared Error
RMSE	Root-Mean-Square Error
PSNR	Peak Signal-To-Noise Ratio
MAE	Mean Absolute Error
CP	Cross-Correlation Parameter
SSIM	Structure Similarity Index Map
